# Human head models and populational framework for simulating brain stimulations

**DOI:** 10.1038/s41597-025-04886-0

**Published:** 2025-03-27

**Authors:** Taylor A. Berger, Miles Wischnewski, Alexander Opitz, Ivan Alekseichuk

**Affiliations:** 1 Department of Biomedical Engineering, University of Minnesota, Minneapolis, MN USA; 2 Department of Experimental Psychology, University of Groningen, Groningen, the Netherlands; 3 Stephen M. Stahl Center for Psychiatric Neuroscience, Department of Psychiatry and Behavioral Sciences, Northwestern University Feinberg School of Medicine, Chicago, IL USA

**Keywords:** Computational neuroscience, Translational research

## Abstract

Noninvasive brain stimulation (NIBS) is pivotal in studying human brain-behavior relations and treating brain disorders. NIBS effectiveness relies on informed targeting of specific brain regions, a challenge due to anatomical differences between humans. Computational volumetric head modeling can capture individual effects and enable comparison across a population. However, most studies implementing modeling use a single-head model, ignoring morphological variability, potentially skewing interpretation, and realistic precision. We present a comprehensive dataset of 100 realistic head models with variable tissue conductivity values, lead-field matrices, standard-space co-registrations, and quality-assured tissue segmentations to provide a large sample of healthy adult head models with anatomical and tissue variance. Leveraging the Human Connectome Project s1200 release, this dataset powers population head modeling for stimulation target optimization, MEEG source modeling simulations, and advanced meta-analysis of brain stimulation studies. We performed a quality assessment for each head mesh, which included a semi-manual segmentation accuracy correction and finite-element analysis quality measures. This dataset will facilitate brain stimulation developments in academic and clinical research.

## Background & Summary

Non-invasive brain stimulation (NIBS) techniques, such as transcranial magnetic (TMS), direct current (tDCS), and alternating current stimulation (tACS), are essential pillars in the ongoing investigation of the human brain. These techniques can safely probe and modulate neural activity in awake humans. NIBS methods are pivotal tools used to study brain-behavior relationships and have been used to investigate and treat several brain disorders^1–4^. The efficacy of these methods depends on their ability to target specific neurological regions of interest. However, identifying the best region and optimizing stimulation in a given individual or population is challenging due to the interaction between stimulation parameters and individual anatomical properties^5,6^. These interactions, if unaccounted for, can result in unintended stimulation outcomes that reduce the efficacy of the therapeutic approach.

Individual variations from the same NIBS interventions are, in part, driven by differences in individual head anatomy and physiology^7^. Anatomical features such as head size, tissue thickness, and gyrification patterns significantly shape delivered electric fields^8^. Accounting for individual anatomical patterns can guide stimulation protocol development for improved targeting. Computational modeling tools have been created to simulate the effect of NIBS in realistic head models^9,10^. Several open modeling software packages using finite element analysis are publicly available, such as SimNIBS^11^, ROAST^12^, and COMETS^13^. These packages automatically generate realistic head models using individual anatomical magnetic resonance imaging (MRI) scans. Additionally, the packages provide a toolset to interact with these models and simulate NIBS protocols to estimate the stimulation behavior better. Modeling tools can support the *ad hoc* development of precision targeting stimulation protocols or *post hoc* interpretation of one or multiple neuromodulation studies.

Neurophysiological properties, foremost tissue conductivities, also vary across individuals. The conductivity of the cerebrospinal fluid, gray matter, and white matter vary manifold across a population^14–17^. Accounting for these conductivity variations provides better insights into stimulation mechanisms^18^. This can be done at a single model level using formal uncertainty algorithms such as a polynomial chaos expansion approach^19^. For large-scale modeling, complete uncertainty quantification can be too expensive. Assigning each model in a virtual population a pseudo-random set of conductivities can reasonably capture this source of uncertainty and contribute to more informed targeting decisions.

As computation tools’ availability and functionality continue to evolve and our understanding of the implications of individual variations on stimulation grows, the standard of modeling practices used to support experimental studies should also continue to develop. Many studies simulating NIBS have relied on a single exemplary head model. To demonstrate this, we looked at studies that investigated the effects of tDCS and tACS on working memory based on recent meta-analyses^20–22^. Out of the studies published in 2015 or after, which is when electric field modeling became openly available, 24 performed electric field simulations. Most of these studies derived anatomical insights from a single head model of an exemplary individual, while remaining studies used a single brain average template. This default approach ignores the morphological variability in a population, which can significantly skew interpretation and give a false sense of precision. It is worth noting that the most common exemplary individual head is a large adult male head (model Ernie^11^), which raises questions about representation and inclusion in the field.

The limited use of advanced computational modeling practices can be attributed to the resources required to develop and verify even a single finite element method model. Generating new head models from imaging data is time-consuming (e.g., over 2 hours on a high-end desktop PC using a FreeSurfer-based image segmentation method), requiring specific expertise if manual corrections are needed. Manual quality assessments of segmentation and meshing processes are necessary for stable numeric simulations. Thus, developing a representative dataset takes hundreds of hours and requires dedicated computing resources. It is unsurprising that many studies use a single exemplary or template model, missing the variance estimates for their generated electric field distribution^23,24^.

The growing number of available brain stimulation studies opens up another opportunity for computational modeling to contribute to meta-analysis efforts^20–22^. Meta-analysis efforts enable researchers to aggregate data across the domain and develop more precise conclusions on the effects of NIBS paradigms by driving common patterns across multiple studies. With a large dataset, meta-analyses can assess the robustness of findings and make more generalizable conclusions. These efforts, in combination with a large, diverse dataset, would allow for a deeper understanding of the nuances of NIBS outcomes, leading to improved treatment protocols.

**Fig. 1:**
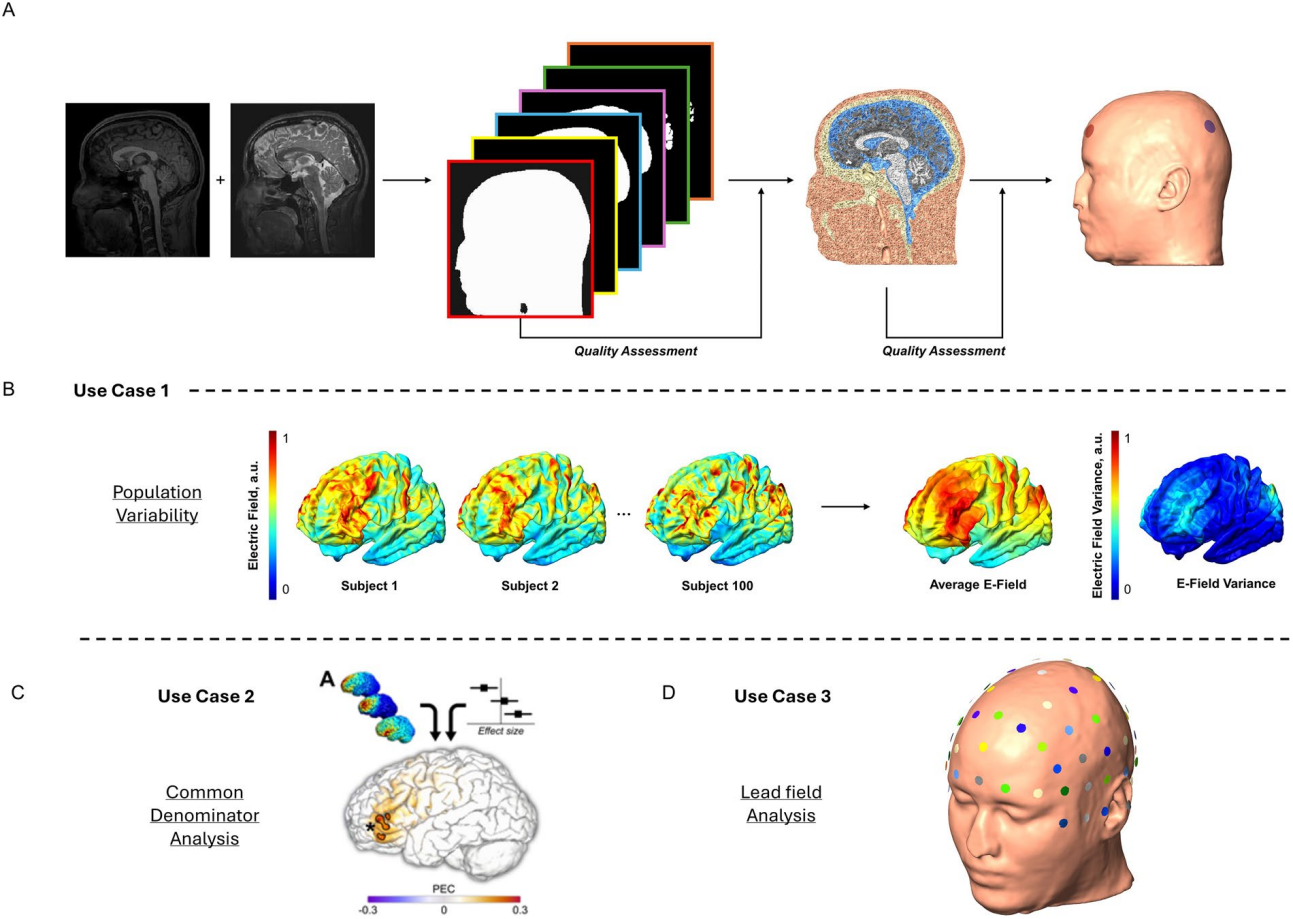
Dataset development and workflow. (**A**) Dataset development workflow using modified SimNIBS 3.2. (**B**) Analysis case 1: Population variability using a single montage. We calculate the average electric field and variance across all subjects for this montage. (**C**) Analysis case 2: Meta-modeling across multiple montages. In this case, a meta-analysis is conducted across multiple montages to derive a “common denominator.” This figure is from Wischnewski ***et al****.*, 2024, with permission. (**D**) Analysis case 3: Lead field analysis to simulate and measure variance in EEG source localization research.

In this data collection, we share an organized computational model dataset for NIBS. This dataset includes 100 preprocessed, quality assured, realistic head models based on imaging data from the Human Connectome Project (HCP) s1200 release^25^. We provide verified finite-element meshes, underlying anatomical images, tissue segmentations, standard space co-registrations, quality metrics, and lead-field matrices. We suggest individual tissue conductivity values for each head model from a range of biologically plausible values^15^. Additionally, we provide straightforward computer code for several use cases of the current dataset. Figure 1 outlines the dataset and potential use cases. This large dataset of realistic head models has multifaceted utility. Here, we present three examples: (1) Demonstration of Population Variability. This dataset can provide insight into the variability of an induced field across a large, diverse population for given stimulation parameters (Fig. 1B). This type of analysis can guide the development of robust targeting stimulation protocols. (2) Common Denominator Analysis. This dataset of 100 head models provides a test bed for meta-modeling techniques. Multiple montages can be easily compared within a standardized space (Fig. 1C). (3) Stimulation Optimization. The provided dataset can assist in robust stimulation protocol optimization efforts, such as in EEG source localization research (Fig. 1D).

In the following sections, we briefly describe the dataset development, the data records, the technical validation, and the sharing and access policy.

## Methods

### Dataset selection

This dataset includes MRI scans from 100 randomly selected, unrelated, healthy young adults (age: 22–35 years, 50 females) from the Human Connectome Project (HCP) s1200 release^25^. HCP subject IDs of participants who fell within the specified age range were put into a single array and randomly permuted in MATLAB 2023. The top 50 males and 50 females were selected to be included in this dataset. We downloaded a T1-weighted MP-RAGE and a T2-weighted MP-SPACE structural MRI for each subject. The image acquisition and preprocessing protocols applied to these images are available with the HCP s1200 release^26^. Notably, HCP applies facial and ear region masking to protect participant privacy, which could potentially influence the accuracy of nasion coordinates. Such influence would possibly lead to minor and normally distributed errors in scalp coordinates across the database. HCP makes the anatomical MRI scans used in this dataset publicly available. Although HCP obtained consent for participation, no further consent was necessary to be included in this dataset.

### Constructing volumetric head models

**Fig. 2:**
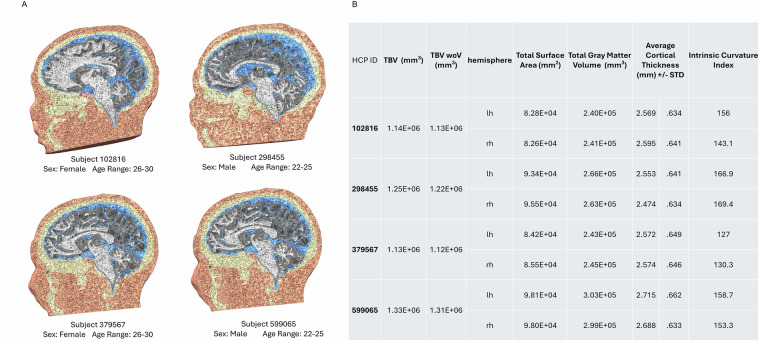
Example subject mesh. (**A**) Four randomly selected subjects (two female, two male) from the dataset. Each mesh has six distinct tissue types (skin, skull, CSF, eyes, GM, and WM). Note that the subjects are not visualized to scale. (**B**) For each subject, the total brain volume (TBV) and the TBV without ventricles (TBV woV) for the whole brain mesh were calculated. Additionally, the total surface area, total gray matter volume, average cortical thickness, and intrinsic curvature index were calculated for each hemisphere.

We used the SimNIBS 3.2.6 *headreco* pipeline^7^ to generate individual realistic volumetric head models. This framework performs automatic tissue segmentation and surface reconstruction using SPM12^27^ and CAT12^28^. The resulting segmentations and surfaces were meshed into FEM models using GMSH 4.9.5^29^. Each model consisted of ~5 to 6 million tetrahedra and six distinct tissue types: skin, skull, cerebral spinal fluid (CSF), eyes, gray matter (GM), and white matter (WM). The meshes and segmentations were inspected manually at every step for anatomical accuracy and mesh degeneracy and corrected, if necessary. If any meshes or segmentations were uncorrectable using straightforward procedures (which occurred four times), we replaced them by selecting the next subject ID of the required gender from the randomly permuted list. For compatibility with new SimNIBS 4.*x* simulations, head meshes provided here originally for SimNIBS 3.2 can be converted using the *convert_3_to_4* function built in SimNIBS 4. We randomly selected four subjects to highlight these individual variations, as shown in Fig. 2A. In each mesh, distinct tissue types are visible and differently distributed for each subject. Even across this small sub-sample of subjects, the statistical characteristics of each volumetric head model varied, see Fig. 2B. Analyzing each mesh, the total brain volume (TBV) fluctuated by 1.97E + 5 mm^3^, and the TBV without ventricles (TBV woV) fluctuated by 1.84E + 5 mm^3^. Looking at each hemisphere individually for features such as total surface area, gray matter volume, cortical thickness, and intrinsic curvature index indicates diversity within the dataset (features are provided with the dataset). All parameters per each head model are included in the dataset and can be used, among other things, as covariates for data analysis. Notably, demographic factors such as age systematically influence head anatomy^30^. Future expansions of this database to include specific sub-populations, such as older adults, would be a valuable step toward supporting the growing number of brain stimulation studies targeting these groups (e.g., elderly).

### Tissue conductivity assignment

All modeling software assign each tissue type a standardized, population-average conductivity value by default. The SimNIBS pipeline’s default conductivities are σ_skull_ = 0.01 S/m, σ_skin_ = 0.465 S/m, σ_CSF_ = 1.66 S/m, σ_eye_ = 0.5 S/m, σ_GM_ = 0.275 S/m, and σ_WM_ = 0.14 S/m^31,32^. To account for realistic biological variability within the dataset, we assigned each subject a unique set of conductivity values from a distribution representative of healthy tissue properties^15^. We pseudo-randomly pulled scalp, skull, and GM tissue conductivity values for each mesh from a Beta(3,3) distribution. The pseudo-randomness here is to ensure that the conductivity values are not correlated across our population. To assess potential correlations between the assigned conductivity values and demographic factors such as age and gender, we performed supplementary analyses. Pearson correlation tests revealed no significant relationships between conductivity values and age for the scalp (r = −0.145, p = 0.151), skull (r = −0.042, p = 0.679), or GM (r = 0.002, p = 0.981). Additionally, a two-sample t-test showed no significant gender differences in conductivity values for the scalp (p = 0.796), skull (p = 0.221), or GM (p = 0.759). Multiple linear regression analysis further indicated that neither age nor gender significantly predicted any re-assigned tissue type conductivity values. The regression model for scalp conductivity explained only 2.1% of the variance (R² = 0.021, p = 0.358), with similarly low R² and p-values for the skull (R² = 0.020, p = 0.368) and gray matter (R² = 0.001, p = 0.950). These results confirm that the assigned conductivity values are not significantly correlated with age or gender, supporting the absence of demographic bias in the pseudo-random assignment process. The CSF, WM, and eye tissue conductivities remained fixed at the default assignments, as these tissues do not significantly impact the simulated electric field^18^. The resulting conductivities used within the dataset are σ_skull_ = 0.002 to 0.03 S/m, σ_skin_ = 0.2 to 0.6 S/m, σ_CSF_ = 1.66 S/m, σ_eye_ = 0.5 S/m, σ_GM_ = 0.1 to 0.6 S/m, and σ_WM_ = 0.14 S/m.

### Lead-field matrices

Lead-field matrices, a powerful tool in neuroimaging and brain stimulation research, are optimized calculations that describe the unique relationship between NIBS techniques and the resulting electric field distribution within a volumetric brain model. By providing detailed insights into how electric fields interact with brain tissues, lead-field matrices enable the individualized placement of electrodes and coils, ultimately aiding in developing effective stimulation protocols. Our dataset provides a lead-field matrix for each individual, calculated using the MKL PARDISO solver. Each unique matrix describes the relationship between electrodes in the 10/10 EEG system^33^ and the individual’s FEM head model. The resulting electric field calculations were interpolated to the middle gray matter (GM) surface, ensuring precise and individualized modeling of brain stimulation effects. The SimNIBS lead-field optimization process is computationally intensive, with a trade-off between resources (up to 12GB per head model) and time (up to 45 minutes per head model). Providing the lead-field for each individual within our dataset increases the accessibility and scope of NIBS modeling by reducing the computational barrier associated with producing viable simulations.

### Standard space Co-registration

Calculating the effects of NIBS on individual FEM models allows for more precise estimations of how NIBS interacts with the brain’s field distributions. Individualized modeling considers unique anatomical properties, such as gyrification, head size, tissue thickness, or conductivities, resulting in a more realistic representation of the brain’s response to stimulation. The main limitation of using individualized FEM models is the difficulty in making direct comparisons across different subjects, as each FEM model is unique. Standard-space co-registration addresses this issue by enabling the interpolation of simulations calculated on realistic head models to standardized spaces like MNI and FreeSurfer Average. This interpolation allows for easier comparison of detailed individual interactions across a larger population, which enhances the ability to generalize findings and develop more universally applicable stimulation protocols. In our dataset, we have included a MATLAB function called *convert_template_to_subject*. This function leverages the features built into the SimNIBS 3.2.6 pipeline to automatically interpolate NIBS simulations from subject-specific space to a specified standard space, facilitating the comparison and generalization of results.

## Data Records

**Fig. 3:**
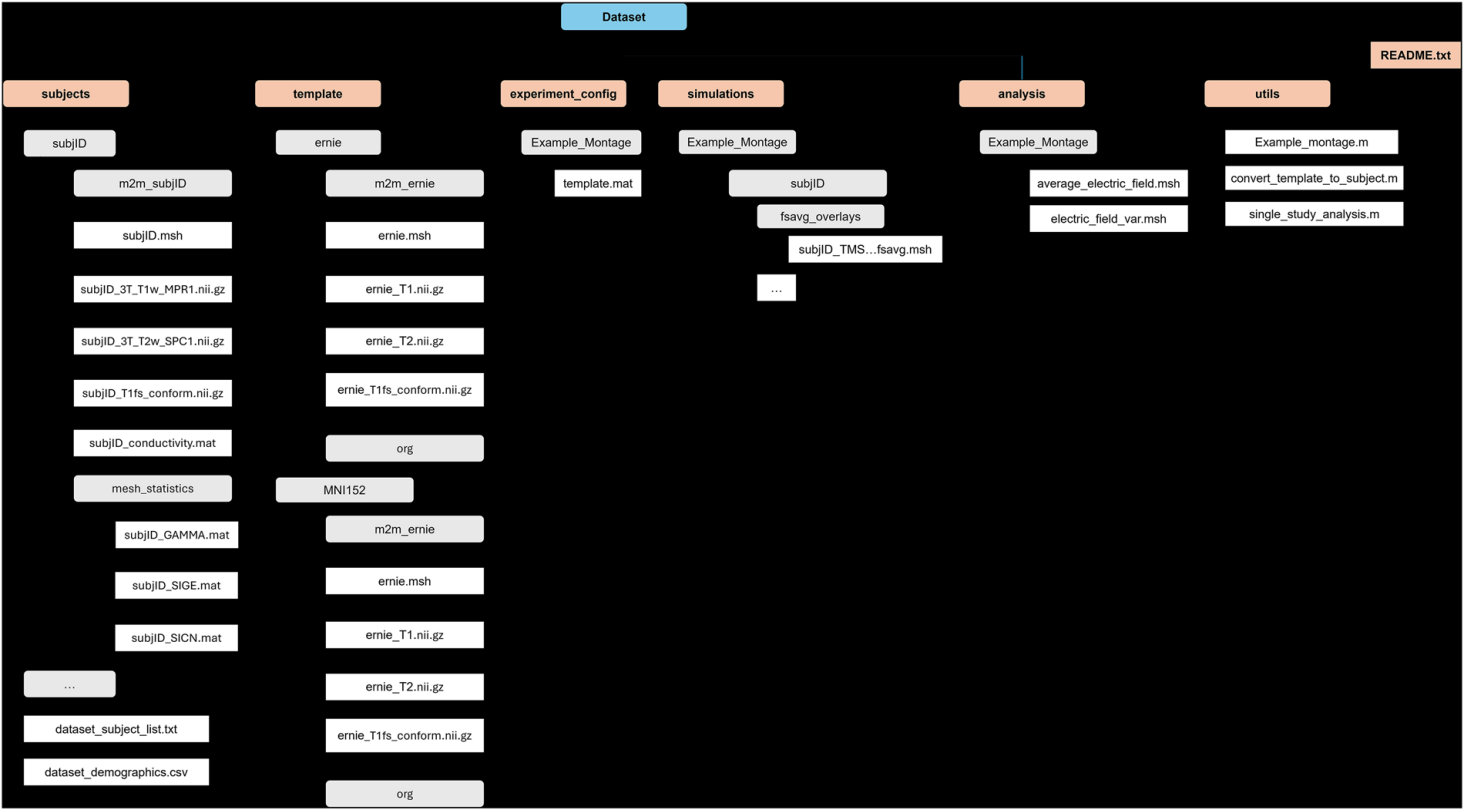
Final dataset structure and naming schemes. There are six main folders within the dataset: (1) subjects, (2) template, (3) experimental_config, (4) simulations, (5) analysis, and (6) utils. Each subject within the dataset exists within its sub-folder within the “subjects” folder. Sub-folders are generated automatically within the support scripts.

The dataset is available on Zenodo^34–40^ (https://zenodo.org/records/13259679). The current dataset includes six folders: “subjects,” “template,” “experiment_config,” “simulations,” “analysis,” and “utils.” It also contains a file named “README.txt.” Detailed descriptions of these folders are provided below, and Fig. 3 illustrates the dataset file structure.

The “subjects” folder contains 100 subfolders named after a subject’s HCP ID. Each subfolder includes T1w and T2w MRI scans, a volumetric head mesh, and other files generated via the headreco pipeline. Each subject folder contains a “mesh_statistics” subfolder with three MATLAB data files containing mesh quality analysis outcomes (see the Technical Validation section for more details). The “subjects” folder also includes two files: “dataset_subject_list.txt,” which lists all subject IDs, and “dataset_demographics.csv,” which provides subject demographics (HCP ID, sex, age).

The “template” folder contains a subfolder for the template subject. Similarly to the dataset subjects, the template subject subfolder includes T1w and T2w MRI scans, a volumetric head mesh, and other files generated via the headreco pipeline. A subfolder named “Example_Montage” is also included in this folder, and it contains a MATLAB data structure named “montage.mat” that sets up the example tACS montage.

The “experiment_config” folder contains a subfolder named “Example_Montage” that contains one MATLAB data structure named “template.mat” that sets up the example tACS montage on the template surface. As simulation montages are generated, subfolders and data structures will auto-populate in this folder.

The “simulations” folder contains subfolders named according to the generated montages. Each montage subfolder contains 100 subfolders named after a subject’s HCP ID. The subject-specific folders contain the results of the SimNIBS simulation pipeline. Specifically, each subject-specific folder contains a subfolder named “fsavg_overlays” that contains a mesh file containing the simulation results interpolated to the FSAverage surface.

The “analysis” folder contains subfolders named according to the generated montages. Each montage subfolder contains two mesh files named “average_electric_field.msh” and “electric_field_var.msh” that contain the electric field averaged across all subjects and the electric field variance, respectively.

The “utils” folder contains three functions used within the electric field simulations. The first file, “example_montage.m,” is a MATLAB script used to configure the parameters within the example montage to the user’s working directory. The “convert_template_to_subject.m” is a MATLAB file that converts the simulation montages from the template subject to each of the 100 HCP subjects. The “single_study_analysis.m” is a MATLAB script that performs a high-level analysis of a single study. We have provided an optional supplementary folder, “utils_simnibs4”^40^, which includes two additional scripts to support conversion from SimNIBS 3 to SimNIBS 4. The first script, “convert_subjects_version.m,” enables the automatic conversion of template subjects and the dataset by utilizing the second script, “run_version_conversion.m,” a backend tool that calls the built-in “convert_3_to_4” function in SimNIBS 4.

## Technical Validation

### Mesh quality assessment

To assess the mesh quality, we conducted a visual/manual and statistical evaluation. We performed two manual inspections on each mesh throughout the meshing process. In the first assessment, we evaluated the tissue segmentation files generated by the SimNIBS 3.2.6 *headreco* pipeline^11^. We visualized each tissue segmentation in FSLeyes 6.0.4^41^ to overlay on the T1w MRI scan. The segmentation was checked for alignment and holes. If corrections had to be made, the segmentations were updated in ITKsnap 3.6. In the second manual inspection, we visualized the 3D volumetric head mesh. If the mesh was discontinuous, we corrected it with the meshFIX 2.1 or AutoDesk MeshMixer 3.5. The surfaces of each tissue were also visually inspected. If the surfaces were disproportionate or degenerate beyond reasonable repair, the mesh was excluded from the dataset and replaced with another random HCP subject’s head image.

**Fig. 4:**
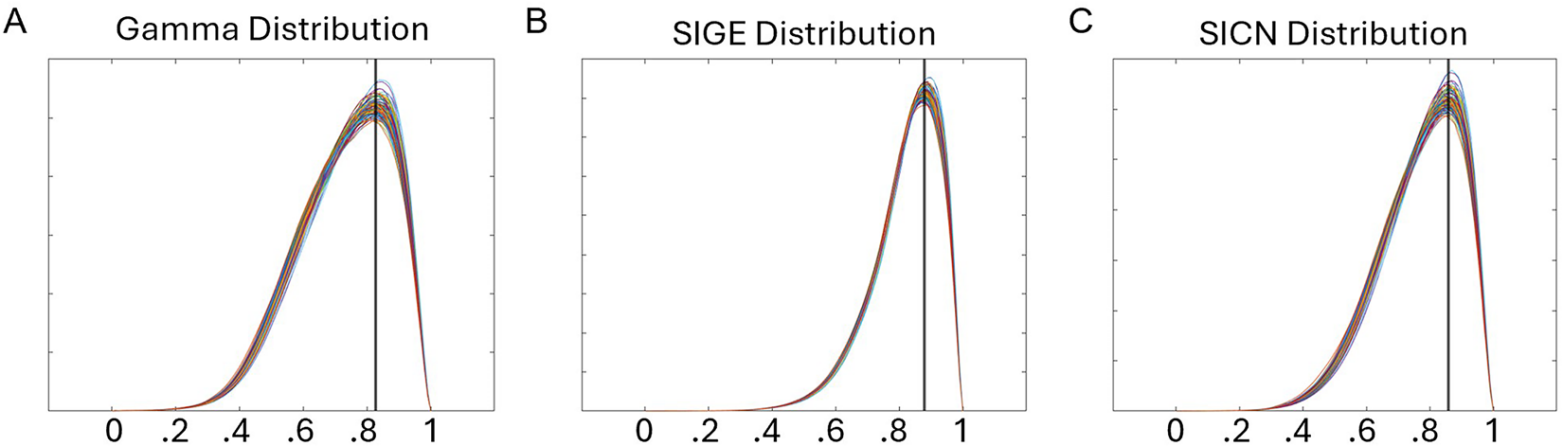
Mesh quality assessment distribution curves for the dataset. The distribution of mesh assessment values for the tetrahedral elements in each volumetric head mesh within the dataset. Each line represents an individual subject’s metric distribution, ranging from 0 to 1. (**A**) Gamma Analysis: The average peak across the entire dataset is 0.8272, visualized as a black vertical line. Approximately 80% of all tetrahedra exhibits Gamma values of 0.60 or higher, indicating the meshes are stable and suitable for reliable simulations. (**B**) Signed Inverse Gradient Error (SIGE) Analysis: The average peak across the entire dataset is 0.8786, visualized as a black vertical line. Approximately 96% of all tetrahedra exhibits SIGE values of 0.60 or higher, indicating the meshes are stable and suitable for reliable simulations. (**C**) Signed Inverse Condition Number (SICN) Analysis: The average peak across the entire dataset is 0.8574, visualized as a black vertical line. Approximately 88% of all tetrahedra exhibit SICN values of 0.60 or higher, indicating the meshes are stable and suitable for reliable simulations.

We used three measurements to quantify the quality of each realistic head model: (1) Gamma, (2) the signed inverse error on the gradient (SIGE) on the FEM model, and (3) the signed inverse condition number (SICN). The distribution curves for each metric are shown in Fig. 4. These metrics are calculated using Gmsh 4.9.5^29^ post-processing tools.

#### Gamma

A FEM mesh is composed of finite elements that discretize the model’s domain. These elements serve as the basis for simulating physical phenomena. Element shape impacts the accuracy, stability, and computational efficiency of simulations. Ideally, finite elements should be close to regular geometric shapes (e.g., a sphere). Poor shape quality, such as flattened or elongated tetrahedrons in 3D meshes, reduces the accuracy and stability of simulations. Gamma is a measurement used to assess the shape quality of the finite elements in a mesh^42^. This metric evaluates how close the element shape is to an ideal shape by comparing the inscribed radius to the circumscribed radius. A high Gamma (close to 1, within the range 0 to 1) indicates an ideal element shape as the inscribed and circumscribed radii are maximally similar. We calculated the Gamma values for each tetrahedron in the volumetric head meshes of all 100 subjects (Fig. 4A). On average, the Gamma value was 0.8272, with the lowest observed peak at 0.8081 and highest at 0.8485. Approximately 80% of all tetrahedra in the meshes had a Gamma value greater than or equal to 0.60, suggesting that the meshes are stable and likely yield reliable simulation results.

#### SIGE

In the simulation process, gradients describe the variation of physical quantities within each finite element. The computation and accurate representation of gradients are essential for understanding and forecasting physical phenomena within a simulated environment. Errors in gradient calculation can lead to significant inaccuracies, particularly near boundaries. Therefore, refining and optimizing the mesh in regions with higher gradient expectations or observations is critical for finite element analysis’s accuracy and stability. SIGE evaluates this by quantifying the inverse of the error of the gradient estimation at each finite element within the mesh. A high SIGE value (close to 1, within the range 0 to 1) signifies less error in gradient approximation, indicating better mesh quality that accurately captures field variable gradients. We calculated the SIGE values for each tetrahedron in the volumetric head meshes of all 100 subjects (Fig. 4B). On average, the peak SIGE value was 0.8786, with the lowest observed peak at 0.8687 and the highest at 0.8990. Approximately 96% of all tetrahedra in the meshes had a SIGE value greater than or equal to 0.60, indicating that the meshes have a consistent quality and can support dependable simulation outcomes.

#### SICN

Gradients across the mesh rely on nodal field values determined by solving a global system of equations derived from discretized governing equations. The system’s sensitivity to numerical errors is determined by its conditioning. A well-conditioned system implies that minor errors in the input data or numerical approximations result in small errors in the solution, indicating stability and reliability. Therefore, monitoring and optimizing the conditioning is crucial for ensuring the accuracy and reliability of FEM simulations. SICN is a measurement used to evaluate the conditioning of the system^42^. A high SICN (close to 1, within the range of 0 to 1) indicates that the mesh is well-structured and that the discretized equations are well-conditioned. A SICN value close to 1 also suggests solution stability and dependability, as small perturbations or errors in the input data are not significantly amplified in the solution process. We calculated the SICN values for each tetrahedron in the volumetric head meshes of all 100 subjects. See Fig. 4C, which plots each subject’s SICN values distribution. On average, the peak SICN value was 0.8574, with the lowest observed peak at 0.8384 and the highest at 0.8788. Approximately 88% of all tetrahedra in the meshes had a SICN value greater than or equal to 0.60, suggesting that the meshes maintain a high quality, likely resulting in accurate simulation results.

### Example electric field montage

**Fig. 5:**
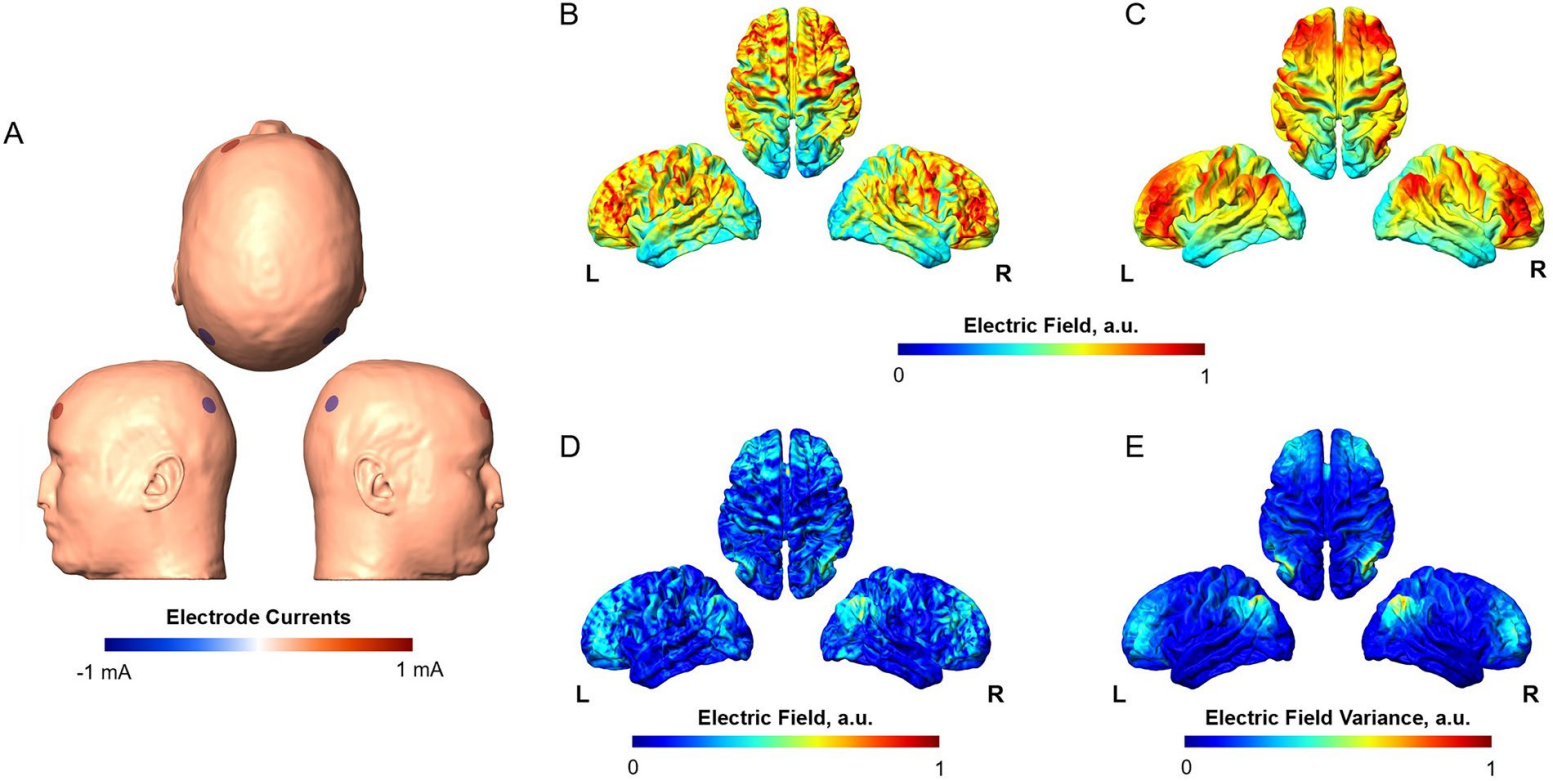
Example montage simulation results and high-level analysis. (**A**) Electrode Montage on the template model following the tACS configuration outlined in Alekseichuk *et al*., 2017. (**B**) Electric field strength on the template model interpolated to the FSAverage surface. (**C**) Average electric field strength across all 100 subjects in the dataset for the example montage on the FSAverage surface. (**D**) The electric field strengths differ between the template model and the average across the dataset. (**E**) Variance in the electric field strength within the dataset.

We modeled an exemplary tACS simulation montage based on a desynchronized montage outlined in Alekseichuk *et al*., 2017. This montage includes four Ag/AgCl electrodes (radius of 1 cm) positioned over AF3, AF4, P3, and P4 according to the international 10-10 system and secured with gel. Stimulation intensity was 1 mA peak-to-baseline. We designed this montage over a template surface (Fig. 5A). The final montage was auto-adapted to all subject-specific models within the dataset. The example montage was simulated within subject-specific space using their uniquely assigned conductivity values for each subject. The results of these simulations were transformed from subject models to the FSAverage surface to facilitate easy cross-subject comparison and evaluation. For a high-level baseline analysis, we computed the average electric field (Fig. 5C) and variance (Fig. 5E) for the simulation across all subjects. The average electric field across the dataset is compared to the electric field calculated on the template surface (Fig. 5B) by calculating the difference between the fields (Fig. 5D). For studies involving multiple montages, these can be synthesized into a meta-model, as demonstrated in our recent work Wischnewski *et al*., 2024.

## Data Availability

Custom codes developed to interact with the dataset are available on Zenodo^34–39^ (https://zenodo.org/records/13259679). The data collection is subdivided into six parts, denoted as “Dataset_1-6”^34–39^. All parts contain the six folders described in the *Data Records* section, with the exception of the “subjects” folder, which has been modified to “subjects_#” corresponding to the number associated with the dataset part. We recommend that all parts be downloaded and merged so that all 100 subjects are within the same subjects folder for optimal use.
